# Head-to-Head Comparison between FDG and ^11^C-Methionine in Multiple Myeloma: A Systematic Review

**DOI:** 10.3390/diagnostics13122009

**Published:** 2023-06-09

**Authors:** Luca Filippi, Viviana Frantellizzi, Paola Bartoletti, Giuseppe De Vincentis, Orazio Schillaci, Laura Evangelista

**Affiliations:** 1 Nuclear Medicine Unit, “Santa Maria Goretti” Hospital, Via Antonio Canova, 04100 Latina, Italy; 2 Department of Radiological Sciences, Oncology and Anatomo-Pathology, Sapienza, University of Rome, 00161 Rome, Italy; viviana.frantellizzi@uniroma1.it (V.F.); giuseppe.devincentis@uniroma1.it (G.D.V.); 3 Nuclear Medicine Unit, Department of Medicine (DIMED), University of Padua, Via Giustiniani, 35128 Padua, Italy; paola.bartoletti@studenti.unipd.it; 4 Department of Biomedicine and Prevention, University Tor Vergata, Viale Oxford 81, 00133 Rome, Italy; orazio.schillaci@uniroma2.it; 5 Department of Biomedical Sciences, Humanitas University, Via Rita Levi Montalcini 4, Pieve Emanuele, 20072 Milan, Italy; laura.evangelista@unipd.it; 6 IRCCS Humanitas Research Hospital, Via Manzoni 56, Rozzano, 20089 Milan, Italy

**Keywords:** multiple myeloma, molecular imaging, PET/CT, amino-acid, choline

## Abstract

The aim of this systematic review is to provide a comprehensive overview of the existing literature, comparing ^18^F-fluorodeoxyglucose (FDG) and ^11^C-methionine (MET) for the imaging of multiple myeloma (MM) with positron emission computed tomography (PET/CT). Relevant studies published from 2013 up to March 2023 were selected by searching Scopus, PubMed, and Web of Science. Selected imaging studies were analyzed using a modified version of the critical Appraisal Skills Programme (CASP). Ten studies encompassing 335 patients were selected. On a patient-based analysis, MET sensitivity ranged between 75.6% and 100%, resulting higher than that measured for FDG (0–100%). MET outperformed FDG for the detection of focal lesions, diffuse bone marrow involvement and mixed patterns. PET-derived parameters resulted higher for MET than for FDG, with a strong correlation with clinical variables (e.g., monoclonal component and beta-2-microglobulin levels, bone marrow infiltration, etc.), although FDG maintained a prognostic impact on outcome prediction. When compared to other tracers or imaging modalities, MET showed stronger correlation and inter-observer agreement than FDG. Although biased by the small cohorts and requiring confirmation through multicenter studies, preliminary findings suggest that MET–PET should be preferred to FDG for PET imaging of MM, or alternatively used as a complementary imaging modality. Some issues, such as tracer availability and the role of MET with respect to other emerging tracers (i.e., ^68^Ga-pentixafor, ^18^F-FACBC and ^18^F-FET), should be the topic of further investigations.

## 1. Introduction

Multiple myeloma (MM) represents a complex and life-threatening condition, with a wide spectrum of manifestation mainly due to the activation of two distinct pathological pathways. The first is represented by the production and accumulation of monoclonal antibodies that can precipitate in the urine and cause renal failure. The second is the activation of skeletal osteoclasts through the nuclear factor kappa-B ligand (RANKL), leading to the formation of lytic lesions and, as a further step, the appearance of pain, fractures and hypercalcemia [1,2]. Patients with a monoclonal-protein detectable in blood or urine, but without typical clinical features (i.e., hypercalcemia, renal failure, anemia, and bone lesions, namely CRAB), are classified as having a pre-malignant condition called “monoclonal gammopathy of undetermined significance” (MGUS) or “smoldering myeloma” (SSM), according to the levels of the monoclonal-protein and the grade of clonal bone marrow involvement [3].

MM accounts for 0.9% of all cancer cases and is slightly more prevalent in men than in women. In recent years, we have been witnessing a dramatic drop in MM mortality rate mainly thanks to meaningful progresses in clinical management and the implementation of molecularly targeted therapies [4]. However, in spite of the aforementioned advances, MM still remains a severe, incurable disease, deeply impacting patients’ overall quality of life. 

There are two main staging systems for MM: the international staging system (ISS) and the Durie–Salmon system (DSS). DSS takes into account immunoglobulin levels, hemoglobin and calcium concentration and the number of bone lesions to predict MM prognosis, while ISS is based on the serum beta-2 microglobulin (Sβ2M) and albumin, resulting in being easier to be computed than DSS [5]. 

Imaging plays a crucial role in MM assessment. In this regard, the 2019 International Myeloma Working Group (IMWG) has recommended the replacement of conventional skeletal survey (CSS) with whole body cross-sectional imaging modalities, such as computed tomography (CT) and magnetic resonance imaging (MRI) [6]. Notably, IMWG recommends positron emission computed tomography (PET/CT), with ^18^F-fluorodeoxyglucose (FDG), for the assessment of therapy response and the evaluation of minimal residual disease (MRD). Recently, new visual descriptive criteria for PET interpretation (Italian myeloma criteria for PET use or IMPeTUs) have been gaining consensus among clinicians for use in both clinical workflow and multicenter trials [7]. However, both false-negative and false-positive results were reported for FDG PET/CT in MM, due to non-FDG avid lesions or concurrent inflammatory or infective lesions, respectively [8]. 

Based on the above, some radiopharmaceuticals other than FDG have been investigated as potential imaging probes in MM. As an example, radiolabeled choline (^11^C/^18^F-choline), a precursor of the biosynthesis of phospholipids, has been successfully employed in the field, providing better lesion visualization and higher tumor-to-background signal with respect to FDG [9,10]. However, choline presents some limitations, such as the high-physiological tracer uptake in liver that limits the use of hepatic activity as a reference according to the Deauville criteria. 

Since MM was found to over-express L-type amino-acid transporter 1 (LAT1), also in correlation with its grade of proliferation and biological aggressiveness, amino-acid PET with ^11^C-methionine (MET) has been proposed as a possible alternative to FDG in this clinical setting [11,12]. 

The aim of the present systematic review is to provide a comprehensive overview of the existing scientific literature on the comparative studies on MET and FDG PET in MM, outlining the pros and cons of these two imaging modalities also in the light of the more recent technological progresses. 

## 2. Materials and Methods

### 2.1. Search Strategy

A literature search until March 2023 was performed in PubMed, Web of Science and Scopus databases in order to retrieve papers related to the topic, according to the Preferred Reporting Items for Systematic reviews and Meta-analyses (PRISMA) guidelines [13]. The terms used were: “^11^C-methionine” AND “PET” AND “multiple myeloma”. Only comparative head-to-head studies between FDG and MET for PET imaging in MM patients published from 2013 up to March 2023, limited to humans, and in the English language, were selected. Conversely, case reports, review papers, conference proceedings, editorial commentaries, interesting images, and letters to the editor were excluded. 

Two reviewers (L.F., L.E.) conducted the literature search and independently appraised each article using a standard protocol and data extraction. The reference lists of the selected studies were carefully checked to identify any additional relevant literature. 

From each study, extracted data were the following: year and location of the study, sample size, patient populations’ demographic characteristics, primary endpoint, sensitivity and specificity of each tracer (when available). Studies with incomplete technical or clinical data were considered ineligible. 

### 2.2. Quality of the Selected Studies 

Selected imaging studies were analyzed using a modified version of the Critical Appraisal Skills Programme (CASP) (https://casp-uk.net/aboutus, accessed on 27 March 2023) checklist for diagnostic test studies. Critical appraisal was performed by two reviewers (L.F. and L.E.), and discrepancies, if any, were solved by discussion with the other authors. 

## 3. Results

### 3.1. Analysis of the Evidence

**Figure 1:**
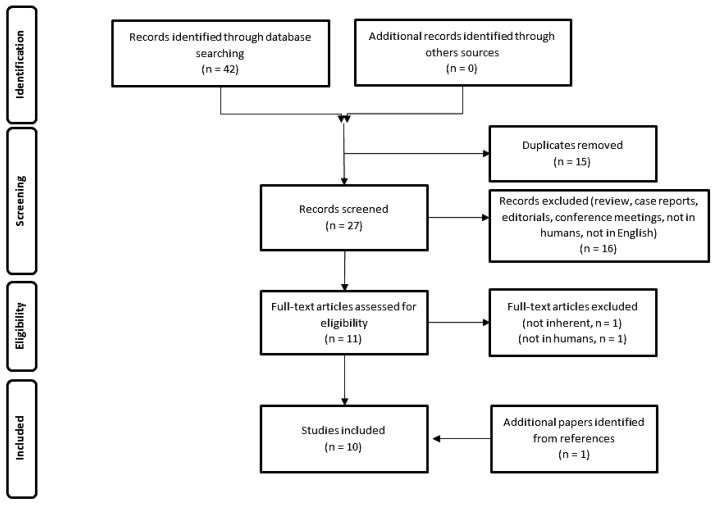
Schematic representation of PRISMA workflow for manuscripts’ selection.

The resulting PRISMA search strategy is shown in Figure 1. From the systematic literature search, 10 papers were selected [14,15,16,17,18,19,20,21,22,23], for an overall number of 335 patients affected by MM or SSM and submitted to both MET and FDG–PET. Table 1 summarizes the main findings of the selected manuscripts. 

From the analysis of the selected manuscripts, three main topics were identified: (1) head-to-head comparison of FDG and MET for detection of newly diagnosed or relapsing MM patients; (2) comparison between FDG and MET-derived PET volumetric parameters in MM diagnosis and prognostic stratification; (3) comparative/ correlative studies of FDG and MET with other radiopharmaceuticals or other imaging modalities (i.e., MRI). 

On a patient-based analysis, MET sensitivity ranged between 75.6% and 100% and resulted higher than that measured for FDG (0–100%), while data on specificity were available in a few studies. With respect to FDG, MET was able to detect more accurate bone marrow involvement and revealed more numerous focal lesions in positive patients [20,22]. Many reasons were correlated with the advantages of MET over FDG. First, a better contrast image of MET than FDG. Second, higher values of qualitative (Deauville score, DS, [22]) and quantitative PET-derived parameters (i.e., SUVmax, SUVmean, SUVpeak) for MET than for FDG [14,20]. Moreover, PET-volumetric parameters measured on MET images showed a stronger correlation with clinical variables, including the grade of bone marrow infiltration [16], than the corresponding values measured on FDG. Nevertheless, this latter tracer maintained a prognostic impact to predict the final outcome in patients with relapsing disease [20,22]. Finally, MET showed better correlation and inter-observer agreement than FDG with respect to other radiopharmaceuticals (^11^C-4DST, ^68^Ga-Pentixafor) or multiparametric MRI [15,19,21]. 

**Figure 2:**
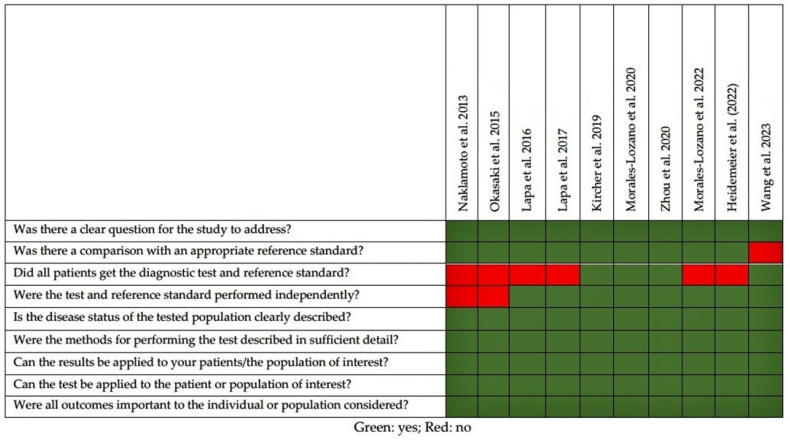
Quality appraisal of selected articles using CASP checklist for diagnostic studies [14,15,16,17,18,19,20,21,22,23].

The quality appraisal of the selected studies is represented in Figure 2. The majority of studies had bone marrow histology or aspiration as a reference, but only in some papers was it carried out in all patients. The most relevant limitations in the selected studies were the following: (1) small sample size, exceeding the threshold of 50 patients only in three studies (30%); (2) the majority (60%) of the selected studies were carried by the same group of research. In this regard, in three manuscripts [17,18,22] the authors included in their cohort, patients were collected from previously published reports.

The main findings of the selected manuscripts for each of the aforementioned thematic areas are summarized in the following paragraphs. 

### 3.2. FDG and MET PET Head-to-Head Comparison 

In a prospective study carried out by Nakamoto et al. [14], 20 patients (MM = 15, plasmacytoma = 5) underwent both MET and FDG PET/CT within an interval time of 3 weeks, due to staging or restaging. PET images were interpreted by experienced nuclear medicine physicians by visual and quantitative analysis. For visual analysis, a 5-point score ranging between 0 (negative) and 4 (positive) was applied, while for quantitative analysis, a region of interest was placed on the suspected lesion, using CT as a reference, and the standardized uptake value (SUV) was measured. The diagnostic performance of both PET imaging modalities was determined on the basis of histopathology or clinical evolution. Of the 20 enrolled patients, 15 were diagnosed to have MM or active plasmacytoma. On patient-based analysis, in six patients submitted to PET for staging, a discrepancy between MET and FDG was registered in one patient (i.e., MET: true positive, FDG: false negative), while the two radiopharmaceuticals yielded overlapping results in the remaining subjects, although MET revealed more clearly the pathological lesions. In the 14 subjects who underwent PET/CT due to restaging, one case presented multiple pathological lesions on MET while it was read as equivocal on FDG–PET. The remaining subjects had consistent results on the 2 PET datasets, although the number of detected lesions was higher on MET than on FDG PET/CT scan. Notably, the grade of tracer uptake resulted higher on MET than on FDG images.

Lapa et al. enrolled 43 patients with newly diagnosed (*n* = 11) or previously treated (*n* = 32) MM, and submitted to both PET/CT with FDG and MET [16]. In all cases, images were interpreted both visually and quantitatively; in particular, a region of interest was drawn on the posterior iliac spine in order to correlate tracer uptake (SUVmax, SUVmean) and the grade of bone marrow involvement. Notably, in some patients, imaging findings were also compared with LAT1 expression, determined on a biopsy specimen obtained from the iliac crest. MET detected positive MM lesions in 90.7% of patients, while FDG resulted positive in 76.7% of cases. MET outperformed FDG for the detection of extramedullary localizations (27.9% vs. 23.3%, respectively), especially for the diagnosis of lymph node involvement. In patients evaluated for LAT1 expression, samples showed homogeneous expression of the transporter on the cell membrane. A correlation among MET and FDG uptake and the grade of bone marrow infiltration was also registered as stronger for MET than for FDG. The same group of research subsequently published a further investigation, partially re-using the same patients in a bicentric study including 78 patients with MM, SSM and plasmacytoma [17]: MET resulted positive in a greater number of patients with respect to FDG. Notably, in two patients with discordant MET and FDG findings, histology confirmed MET results in both cases. In addition, MET yielded a higher inter-reader agreement in comparison with FDG. 

The biological mechanisms underlying FDG false-negative MM were investigated by Kircher et al. [18]. Nine patients with histology-proven and sierologically active MM, characterized by FDG-negative and MET-positive PET/CT findings, were enrolled and compared with six MM subjects with positive and concordant findings both on FDG and MET PET. Of the 15 patients, 13 had relapsing or therapy-refractory MM while 2 presented a newly diagnosed disease. PET results were correlated with hexokinase 2 (HK-2) expression, determined on bone marrow specimens. In the cohort of FDG-negative patients, MET PET/CT revealed multiple active lesions in all patients, also detecting extramedullary localization in one case. Histology revealed monoclonal plasma cells in all 15 patients and showed intense glucose transporter expression in all cases. Most importantly, the authors did not find any significant differences in HK2 and glucose-6-phosphatase expression between patients with FDG-negative and FDG-positive PET-findings, thus suggesting that the lack of FDG incorporation in MM cells might dependent on some still unknown mechanisms. 

The usefulness of MET PET in patients affected by FDG-occult MM was assessed by Wang et al. [23]. All participants had lytic bone lesions on CT scan, biochemical evidence of MM, no FDG uptake on previously performed PET/CT, and had been previously treated with one or more lines of therapy. All seven patients were submitted to MET that resulted positive in five cases (71.4%), with a SUVmax ranging from 2.8–6.4. In the aforementioned paper, no histology was performed as a confirmation of MET–PET and all MET-avid lesions were considered as pathological. 

### 3.3. Diagnostic and Prognostic Impact of FDG and MET-Derived Volumetric Parameters

The diagnostic value of whole body tumor burden, assessed by both MET and FDG–PET in MM patients, was assessed by Morales-Lozano and coworkers in two distinct studies. In a first analysis [20], the authors enrolled 22 patients with newly diagnosed, therapy-naïve MM, submitted to both MET and FDG–PET. On PET images, aside from SUV-based values (SUVmax, SUVmean, SUVpeak), some volumetric parameters were measured. Total Metabolic Tumor Volume (TMTV) was obtained through the lesions’ segmentation by applying a dedicated algorithm based on a SUVmax-threshold (41%). By multiplying TMTV for SUVmean, the authors also calculated total lesion glycolysis for FDG and total lesion methionine uptake (TLMU) for MET. On a patient-based analysis, all the examined subjects were positive on both MET and FDG imaging. Notably, MET showed a diffuse pattern of bone marrow involvement in a greater number of patients than FDG, indicating that MET may be suitable for the detection of both diffuse infiltration and focal lesions. On a lesion-based analysis, a moderate agreement between MET and FDG was registered (*k* = 0.66), although MET revealed a significantly higher number of focal lesions in 50% of patients. Concerning the quantitative analysis, all SUV-based values (SUVmax, SUVmean, SUVpeak) were higher for MET than for FDG. This trend was also confirmed for volumetric parameters: median TMTV resulted in 443.4, and 134.8 cm^3^ for MET and FDG, respectively, while median TLMU was 2021.4 g, and TLG resulted in 598.4 g. Notably, the authors also assessed the correlation among some PET variables and clinical parameters: only a moderate correlation was registered between FDG-based SUVmax and SUVmean and high beta-2 microglobulin levels, while MET-derived volumetric parameters (TMTV and TLMU) showed a high correlation with the levels of monoclonal-component and the severity of bone marrow infiltration. Table 2 summarizes the results of quantitative analysis carried out in the previously cited paper.

In another more recently published paper from the same group [22], 52 patients (44 MM, 8 with SMM) were enrolled. FDG and MET were employed both for staging/restaging and the prediction of patients’ outcome. Of the enrolled patients, 18 were affected by newly diagnosed MM (NDMM) and the remaining had pre-treated MM. On a patient-based analysis, 46 cases showed concordant MET and FDG findings (44 as positive and 2 as negative), while 6 were MET-positive and FDG-negative, with a weak correlation between the two tracers (*k* = 0.361). Of note, in 64% of patients with concordant positive MET and FDG findings, the number of detected focal lesions was greater on MET. The authors also assessed the pattern of bone involvement identified by the two tracers; with respect to FDG, MET revealed diffuse bone marrow infiltration in a greater number of cases and also detected a more relevant number of mixed patterns (diffuse BM uptake plus focal lesions). On a lesion-based analysis, MET identified a greater number of focal lesions and, most importantly, when lesions were scored according to the DS criteria, a major number of focal lesions were classified as DS 4 on MET with respect to FDG. It has to be underlined that tumor-to-background ratio calculated on spine, and iliac bone on MET images, correlated with the grade of bone marrow infiltration, while this relationship was not found for FDG. Concerning the prognostic impact of PET-derived parameters in the relapsed cohort of MM patients, the following FDG parameters were associated with poor prognosis: more than 3 focal lesions, more than 10 focal lesions, median (p50) TMTV and 75th percentile (p75) TMTV. Regarding MET parameters, TMTV p50/p75 and TLMU p50/p75 were correlated with shorter progression-free survival.

### 3.4. Comparison of FDG and MET with Other Radiopharmaceuticals or Other Imaging Modalities (MRI)

Okasaki and coworkers evaluated the diagnostic performance of MET, FDG and [methyl-11C] 40-thiothymidine (^11^C-4DST); this latter tracer was employed as a surrogate biomarker of DNA synthesis in 64 patients with MM or MGUS (newly diagnosed = 21, restaging = 43) [15]. Patients were enrolled into two distinct studies: study one enrolled subjects presenting lytic lesions, easily detectable on CT; and study two considered subjects with diffuse or normal CT findings, who underwent bone marrow aspiration within 1 week after the execution of the three PET/CT scans. Images were evaluated both visually and quantitatively. The number of equivocal findings was higher for FDG than for MET and ^11^C-4DST; in particular, both MET and ^11^C-4DST showed a superior detection rate for lesions located in the skull, thanks to the lack of brain uptake. Notably, there was a correlation among positive tracers’ uptake and MM involvement of bone marrow, which was more significant for MET and ^11^C-4DST. Although the positive/negative predictive value and accuracy were not statistically different among the three tracers, the area under the ROC curve was greater for MET and ^11^C-4DST with respect to FDG.

An interesting study compared the diagnostic performance of multi-parametric (mp) whole body (WB)-MRI for medullary lesions’ detection with respect to MET and FDG–PET [21]. An overall number of 44 (newly diagnosed *n* = 3, progressive disease *n* = 2, response evaluation *n* = 39) MM patients who underwent FDG and mpWB MRI for staging or response assessment, within a 10-day interval, were included. Of the enrolled subjects, MET–PET was also available for comparison in 24 cases. The following criteria were identified to categorize mpWB MRI as positive: fat fraction < 20%, diffusion-restriction, and hypointensity on the T2-weighted images. Concerning MET and FDG images, aside from qualitative analysis, the authors extracted the following parameters: SUVmax, SUVmean, SUVpeak. In addition, PET-images were scored according to DS criteria. In 37 cases, bone marrow biopsy was available as a reference. An excellent grade of agreement was registered among MRI-readers for the classification of medullary lesions as vital/non-vital. Notably, 94.1% of lesions categorized as vital on the basis of MRI also received a DS4 or DS5 on FDG and MET images. However, it has to be underlined that by comparing mpWB MRI and PET findings, the interobserver agreement was fair (k = 0.53) and strong (*k* = 0.79) for FDG and MET, respectively. 

FDG and MET were both compared with ^68^Ga-Pentixafor, a radiopharmaceutical targeting chemokine (C-X-C motif) receptor 4 (CXCR4), for the imaging of SSM in a retrospective analysis carried out by Zhou and colleagues [19]. Ten patients submitted to triple tracer PET/CT with MET/FDG/^68^Ga-Pentixafor were enrolled. Images of each PET imaging modality were analyzed both qualitatively and quantitatively: a ROI was placed in the center of each L2-L4 vertebra, and maximum and mean tumor-to-background ratios (TBRmax and TBRmean) were calculated by comparing vertebral SUVmax and SUVmean with the SUVmean measured in the right atrium. SUVmean and TBRmean measured in L2-L4 on MET and ^68^Ga-Pentixafor correlated with bone marrow (BM) plasma cell (PC) infiltration, while this relationship was not found for FDG. 

## 4. Discussion

From the analysis of the selected papers, some considerations about the role of FDG and MET for the imaging of MM can be made. MET outperformed FDG in terms of sensitivity and diagnostic accuracy for the PET imaging of MM at staging and for the detection of a viable tumor after therapy. In particular, MET was more capable to accurately identify MM extramedullary localizations and define the grade of bone marrow involvement, with a strong correlation with the histological findings. Notably, overall tumor burden determined on MET images showed a strong correlation with clinical variables and impacted on patients’ prognosis, thus reflecting the severity of MM involvement [20,22]. 

However, in spite of these promising results and although it can be prepared “in house” and employed for clinical use, insofar, as it has been established by current pharmacopeia (http://www.pharmacopeia.cn/v29240/usp29nf24s0_m13060.html, accessed on 27 March 2023), MET did not find a widespread application in clinical practice due to several issues. First of all, the short half-life of ^11^C limits the use of these tracers to the PET centers equipped with an in situ cyclotron. Second, ^11^C-radionuclide has a suboptimal positron energy for PET imaging, thus negatively impacting on image quality and spatial resolution [24]. Third, in a single day, the synthesis of MET is limited, therefore guaranteeing the examination in a small number of patients. Fourth, MET accumulates in normal bone marrow, and in the liver and pancreas, thus limiting the accuracy in case of faint or mild invasion. In this regard, a synthetic amino-acid labeled with ^18^F-fluorine, namely ^18^F-fluciclovine or ^18^F-FACBC [25], recently implemented in clinical practice for the imaging of prostate cancer recurrence, provided promising results in a preliminary study carried out in patients with newly diagnosed MM [26]. On the same path, Czyż and coworkers assessed the potential of ^18^F-ethyl-tyrosine (^18^F-FET), another amino-acid tracer used in neuro-oncology, in 32 MM patients; although it was not compared to FDG, ^18^F-FET showed good diagnostic performance, detecting a greater number of lesions with respect to CT in subjects with a newly diagnosed disease [27]. 

It has to be underlined that, although MET showed superior diagnostic performance with respect to FDG for MM staging and post-treatment evaluation, FDG maintained a prognostic impact to predict survival in relapsing patients and correlated with high-risk cytogenetic [22,28,29]. It is worth mentioning that MET and FDG provide different and complementary information since they reflect distinct metabolic pathways. On the one hand, FDG has a well-established role in oncology and has been widely employed, alone, or in combination with other tracers, as imaging biomarker of tumor aggressiveness and dedifferentiation [30,31]. However, it is well known that some tumors do not exhibit FDG-avidity due to several, and still, not completely understood reasons (lack or reduced expression of glucose transporters or hexokinase, increased expression of glucose-6-phosphatase, etc.) [32]. On the other hand, amino-acid PET exploits the overexpression of (predominantly L-type) AA transporters in several types of malignancies. MET has been widely employed for the imaging of brain tumors but its applications in other clinical fields are still not fully investigated [33]. Therefore, it might be hypothesized that the combined use of the two tracers in well-selected patients, in order to simultaneously obtain diagnostic and prognostic information following the procedure, can be applied for prostate cancer and neuroendocrine tumors [34,35]. Indeed, being about 10–15% of MM patients without FDG avidity, MET could complementary recognize viable tissues at baseline and after therapy. In this perspective, the implementation of highly performing technologies, such as digital PET or long axial-field-of-view PET/CT scanners, might have a role to perform dual tracer PET/CT studies with low-dose and fast protocols, thus limiting patients’ radiation burden [36].

An interesting role for MET would be found in the identification of “high-risk” SMM rather than FDG; indeed, both Zhou et al. and Lapa et al. [17,19] described the higher sensitivity of MET than FDG in detecting bone marrow infiltration also in this setting. 

**Figure 3:**
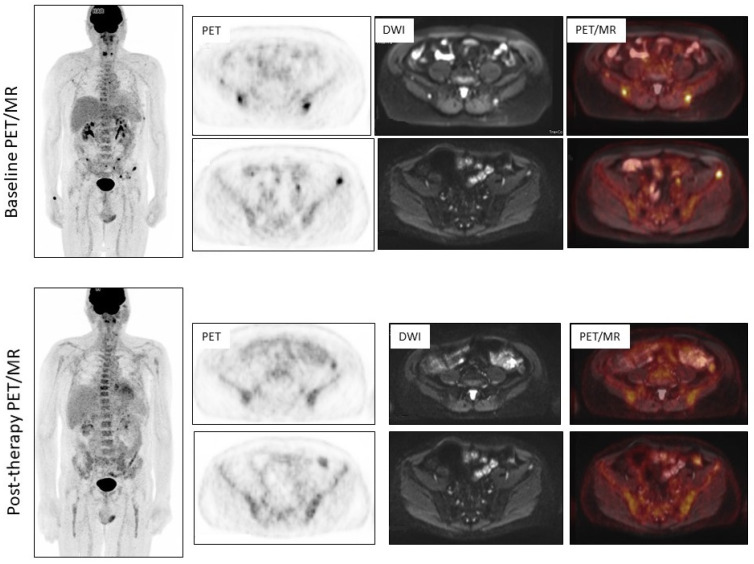
60-year-old male. Pathological fracture in C6. Bone marrow biopsy was compatible with multiple myeloma. (**upper row**) Baseline FDG PET/MR demonstrated the presence of multiple focal uptake in the bones, without extranodal involvement, corresponding to an increase of signal at DWI images. After induction chemotherapy (**lower row**), FDG PET/MR demonstrated no uptake in the site of pre-existing lesions, with the persistence of a slight signal at DWI images. Therefore, FDG–PET was conclusive for the presence of non-viable tissue.

None of the selected manuscripts employed hybrid PET/MRI for MET and FDG imaging [37]. The combination of FDG–PET and MRI was found to detect more bone marrow metastases than either imaging modality alone, and might have a great potential for MM imaging and prognostication [38]. This topic should be the object of future investigations. Figure 3 depicts a clinical case from authors’ series illustrating the usefulness of FDG PET/MRI in MM staging and restaging.

Finally, MM cannot be left behind in the so-called “theranostic revolution”. Theranostics combines diagnosis and therapy in a unique approach, since it involves the sequential administration of a couple of identical or very similar radiopharmaceuticals, both targeting a specific tumor-associated biomarker; the first one labeled with a nuclide suitable for imaging with PET or gamma-camera, the second one conjugated with a nuclide emitting beta or alpha particles to exert anti-tumor effects [39,40,41]. In this perspective, C-X-C-motif chemokine receptor 4 (CXCR4) has emerged as a relevant theranostic biomarker in MM and other malignancies, and the theranostic couple “^68^Ga-pentixafor/^177^Lu-pentixather” holds promise to move the field forward [42,43]. However, this approach is still far from passing from “bench-to-bedside” and its role with respect to metabolic imaging, with FDG and MET, should be deepened by further studies. 

## 5. Conclusions

Preliminary results emerging from the analysis of the existing literature, although biased by the small cohorts of the included studies and requiring further confirmation through multicenter cooperations, suggest that MET should be preferred to FDG for the assessment of MM patients at staging and after therapy, although FDG might be considered in well-selected, high-risk subjects for prognostic stratification. Some issues, concerning tracer availability and its role with respect to other emerging imaging modalities, should be addressed by further investigations. 

## Figures and Tables

**Table 1:** Main findings of the selected papers on the applications of FDG and MET–PET in MM.

Authors	Country	Year of Pub	N of pts	Median Age (Range)	Male/ Female	Primary End-point	FDG/MET Sensitivity	FDG/MET Specificity	Comments
Nakamoto et al. [14]	Japan	2013	20	(45–80)	11/9	Compare FDG with MET in MM	78%/89% *	100%/100% *	MET revealed more lesions than FDG. MET is useful in case of inconclusive FDG
Okasaki et al. [15]	Japan	2015	64	(33–84)	40/24	Compare FDG and MET with ^11^C-4DST in detection of bone marrow in MM	60/86.7% *	76.1%/76.1%	MET can detect more active lesions than FDG in bone marrow
Lapa et al. [16]	Germany	2016	43	(39–82)	24/19	Compare FDG and MET in staging and restaging of MM	76.7%/90.7% *	-	MET can detect more lesions than FDG both in medullary and extramedullary MM lesions
Lapa et al. [17]	Germany	2017	78 (4 SP, 5 SSM and 69 sMM)	(31–76)	55/33	The superiority of MET than FDG in MM	60.3%/75.6% *	-	MET can detect more lesions than FDG MET can detect more viable tissue than FDG
Kircher et al. [18]	Germany	2019	15	(51–73)	10/5	To understand the biological reason for the negative FDG PET in viable MM by using MET	60%/100% *	-	No differences in HK2 expression was found among FDG-negative and FDG-positive MM. Other reasons can be linked with this finding
Zhou et al. [19]	Germany	2020	10	(41–74)	8/2	To explore the role of FDG, MET and ^68^Ga-Pentixafor PET/CT in SMM	-	-	MET and 68Ga-Pentixafor PET/CT demonstrate higher sensitivity than FDG PET/CT in detecting bone marrow involvement in SMM.
Morales-Lozano et al. [20]	Spain/Germany	2020	22	(37–79)	14/6	To compare MET suitability for the assessment of metabolic tumor burden in comparison to FDG PET.	100%/100% *	-	MET seems to be a more sensitive and accurate surrogate for total myeloma burden as compared to FDG.
Heidemeier et al. [21]	Germany	2022	24	(49–78)	-	To identify a new wbMRI algorithm for bone marrow lesions, by using FDG and MET PET	-	-	DWI + CSI and FF + T2 sequences can help in identifying MM lesions
Morales-Lozano et al. [22]	Spain/Germany	2022	8 SMM, 44 MM	61 (37–83)	28/24	To compare MET with FDG PET/CT in SMM and MM	84.6–96.1%	-	FDG has a prognostic role in MM MET can detect more lesions than FDG TMTV and TLMU at MET PET show a prognostic meaning
Wang et al. [23]	USA	2023	7	(58–93)	5/2	MET PET role in case of FDG negative scan	0%/80%	0%/100%	MET PET can be used in case of FDG negative scan for detecting focal avid lesions in MM patients

* patient-based analysis; SP = solitary plasmocitoma; SSM = smouldering myeloma; SMM = symptomatic multiple myeloma; DWI = diffusion weighted imaging; CSI = chemical shift imaging; FF = fat fraction; TMTV = total metabolic tumor volume; TLMU = total lesion MET uptake.

**Table 2:** Quantitative parameters of MET and FDG–PET and their correlation with clinical variables.

Parameter	FDG (Median and IQR)	Corr. with Clinical Variables	MET (Median and IQR)	Corr. with Clinical Variables
SUVmax	8.76 (3.45–62.23)	beta-2-micro.	16.40 (6–195.6)	-
SUVmean	3.55 (1.82–7.74)	beta-2-micro.	4.59 (2.79–8.35)	-
SUVpeak	6.56 (2.82–39.85)	beta-2-micro.	10.72 (4.64–126.50)	-
TMTV	134.8 cm^3^ (5.6–524.9)	beta-2-micro.	443.4 cm^3^ (145.2–1102.6)	beta-2-micro, M-component, BM infiltration
TLG	598.4 g, (10.7–2086.4)	-		
TLMU	-		2021.4 g (761.6–6061.4)	beta-2-micro, M-component, BM infiltration

SUVmax = maximum standardized uptake value; SUVmean = mean standardized uptake value; SUVpeak = peak of standardized uptake value; beta-2-micro. = beta-2-microgloblulin; IQR = interquartile range; corr. = correlation; TMTV = total metabolic tumor volume; TLMU = total lesion MET uptake, TLG: total lesion glycolysis.
